# Intestinal helminthiasis, anaemia and associated risk factors in a cross-section of the population of Melong, Littorial Region of Cameroon

**DOI:** 10.1038/s41598-023-46446-9

**Published:** 2024-01-05

**Authors:** Haman Katamssadan Tofel, Lem Edith Abongwa, Ruth Fri Ndifor, Helen Ngum Ntonifor

**Affiliations:** 1 Department of Biological Sciences, Faculty of Science, The University of Bamenda, BP 39, Bambili, North West Region Cameroon; 2 African Centre of Excellence for Genomics of Infectious Diseases (ACEGID), Redeemer’s University, Ede, Osun State Nigeria

**Keywords:** Microbiology, Medical research, Pathogenesis

## Abstract

Assessment of risk factors of intestinal helminths and anaemia in various geographical regions is important for the development of appropriate control strategies. This study aimed at determining the risk factors associated with intestinal helminths and anaemia in Melong, Cameroon. A total of 325 participants were recruited in this study between September–December 2021. Faecal samples were examined using the formol-ether sedimentation technique while haemoglobin level was measured using a HemoCue spectrometer. Data on demographic and behavioural factors was collected and used to determine the risk factors using a pretested structured questionnaire and analysed using SPSS. The overall prevalence of intestinal helminths was 24.6% (80/325). Helminths recovered from the study included hookworm (16%; 52/325)*, Schistosoma mansoni* (10.8% 35/325*) Ascaris lumbricoides* (1.9%; 6/325), and *Trichuris trichiura* (0.6%; 2/325) with 15 participants having multiple infections (4.6%). The geometric mean egg density was 77epg and ranged from 20 to 560 epg of faeces. Males and age group ≤ 5 years had the highest parasite intensity (248epg). The overall prevalence of anaemia was 33.5% (109/325). Anaemia was significantly (p = 0.001) high at 48.8% (39/80) in those infected with intestinal parasites compared to non-infected individuals. Age group of 31–45 years; [3.42(1.05–11.21)] and > 65 years [6.21(1.75–12.47)], poor knowledge [0.41(0.67–6.07)], no regular deworming [0.70(1.76–21.96)], mud floors toilet [6.18(1.61–23.79)], toilets made of sticks [16.5(4.24–64.31)], and participants who did not have stomach/abdominal pains [0.22(0.07–0.67)] were significant predictors of helminth infections. Age group < 15 years [2.58(1.09–6.11)], geophagia [3.69(1.91–9.33)], hookworm infection [3.26(1.49–7.11)], *S. mansoni* [1.72 (1.16–3.41)] and those with multiple infections [1.76(1.04–2.88)] were identified as risk factors for anaemia. Risk factors identified in this study can be used to improve the control mechanism put in place by the government.

## Introduction

Intestinal helminth infections are among the major health problems throughout the World, particularly in developing countries^1^. Over one-third of the world's human population have diseases caused by soil-transmitted helminths (intestinal parasitic nematode worms) which include *Ascaris lumbricoides*, whipworm (*Trichuris trichiura*), and hookworm (*Necator americanus*/ *Ancylostoma duodenale*) occurring mainly in low and middle-income countries of Asia, Africa, and Latin America^2^. Collectively, soil-transmitted helminths (STHs) are the most common of the 20 major neglected tropical diseases and the most widespread chronic infections globally^1^. STHs infect the host after ingestion of undercooked food, walking barefooted, when hands contaminated with soil are put in the mouth, or through mechanical fly vectors^2,3^. Many individuals harbour more than one STH concurrently, particularly in rural communities lacking basic hygiene and sanitation^4^. Furthermore, intestinal schistosomiasis which is caused by *Schistosoma* species (*S. guineensis, S. intercalatum, S. mansoni, S. japonicum,* and *S. mekongi*) is a water-transmitted helminth and infects humans by penetrating the skin actively when a man comes in contact with water infested with schistosome cercariae.

Record from World Health Organization^5^ shows that more than 1.5 billion people, or 24% of the world's population, are infected with helminths and 240 million others are infected with schistosomiasis. Of this, more than 613 million school-age children in the world are at risk of STHs infections^6^. Morbidity induced by infection with the major intestinal helminths results in an estimated 5.19 million disability-adjusted life years^7^. Infections are widely distributed in tropical and subtropical areas, with the greatest numbers occurring in sub-Saharan Africa, the Americas, China, and East Asia where co-infection with Schistosomes and STHs is common^5^. This prevalence varies from one study to another due to different risk factors which include age, toilet types, walking barefoot, and the habit of not washing hands before meals^8,9^.

Intestinal helminths are endemic in Cameroon with rural areas being more infected than urban areas^10,11^. In Cameroon, the prevalence of intestinal worms ranges from 17.6% to 29.1% across the different regions^4,12,13^ with approximately 7.6 million children at risk of STH infections^5^. The prevalence of schistosomiasis in the country ranges from 31.5 to 43.0% and rural communities are the most affected. Species of Schistosomes endemic in Cameroon include *S. haematobium, S. mansoni* and *S. intercalatum*^14,15^.

Anaemia is regarded worldwide as a medical condition deserving sustained public health intervention and affects 1.62 billion people globally^16^. Anaemia is mostly prevalent in the developing world and is associated with parasitic infections such as hookworm infection, schistosomiasis, and malaria which contribute most to anaemia^17,18^. Previous studies showed that the prevalence of anaemia associated with helminths ranged from 10.3–27.1%^12,17,19,20^ and about 27.1% in the Cameroon^11^. Equally, anaemia in school-aged children is strongly associated with moderate to heavy hookworm infections^15,19,21^. Anaemia has many irreversible impacts: it impairs physical growth, impairs immune function, increases susceptibility to infections, and weakens motor development leading to reduced cognitive ability and short or long-term effects including mortality in severe cases^20^.

In this study, we investigated the prevalence and associated risk factors of intestinal helminthiasis, and anaemia, in a semi-urban area in the Littoral region of Cameroon that acts as a crossroad where passengers stop to eat or buy different foodstuff (fruits, meat, plantain, vegetable, etc.) from various parts of the country. Like any other deprived area in the country, lack of access to improved sanitation facilities and poor hygienic behaviour characterizes the study area.

## Method

### Study design

**Figure 1:**
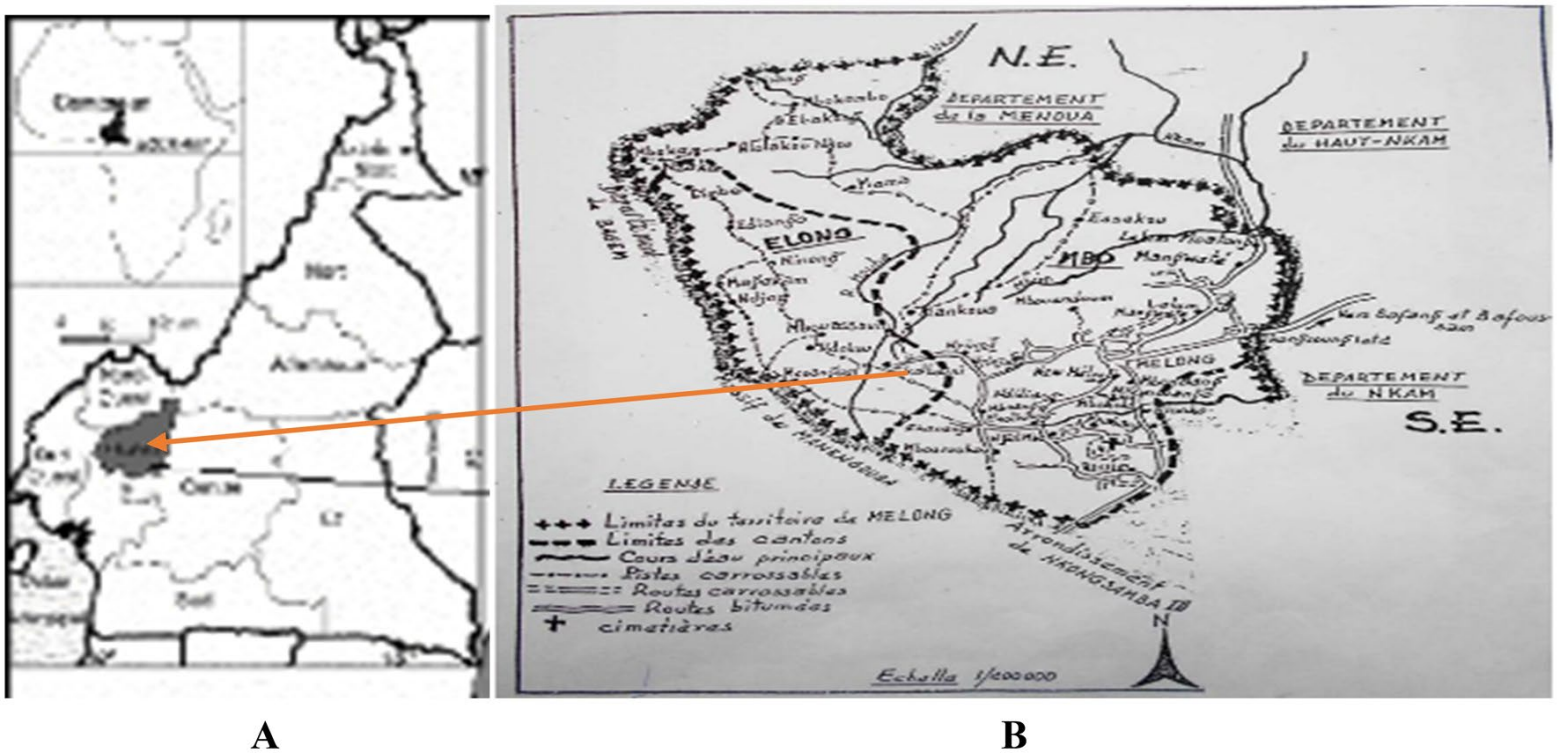
Map of Cameroon (**A**) showing the study area (**B**) (source: Divisional Delegation of Public Health Melong)

This was a community based, cross-sectional study where sample collection was selectively purposive, since the study aimed at targeting a wide age range within the defined study area. The study was carried out between September-December 2021 in Melong, a small community in the Littoral Region of Cameroon (Fig. 1). This period coincided with the end of the rainy season and the beginning of the dry season. Melong is a cross-road from Bamenda to other major towns in Cameroon where travelers stop and consume different types of foods (roasted plantains, yams, cocoyam, potatoes, plumes, soya etc.) and also buy fruits such as mangoes, pineapples and pawpaw that might encourage the propagation of parasites. Stool samples were collected for the detection of the parasites and capillary blood for the measurement of haemoglobin concentration.

### Study site

Melong lies on the N5 (national road 5) road to the North of Nkongsamba with GPS coordinates 5̊ 7ꞌ16ꞌꞌN and 9֠ 57ꞌ10ꞌꞌE and elevation of 790 m (2,590 ft.) above sea level. The terrain is mountainous and has numerous streams which are the major sources of water for the people. It has a characteristic dense equatorial rainforest with very fertile volcanic soils with hills and valleys. The temperature ranges from 17–18 °C at night and from 26–29 °C during the day. The total population of Melon is 37,086 and it is a native population of Mbo’o, a group of “Sawa” people who are established in the Littoral and Southwest regions of the country^22^.

### Ethical considerations

Ethical clearance for the study was obtained from the university of Bamenda ethical committee (2021/100H/UBa/IRB). Participants who were 18 years and above were recruited after signing the informed consent while consent forms for minors (participants less than 18 years) were signed by the guardians or parents. All methods were performed in accordance with the relevant guidelines and regulations of the ethical review board.

### Study population and sample size

All inhabitants of Melong irrespective of their ages and gender were eligible to participate in the study. However, all those with a recent history of antihelminthic treatment (taken two months before the study), and those who were severely sick were excluded from the study. The minimum estimated size was calculated to be 192 participants using the Lorenz formula below;

$$ {\text{N}} = \frac{{(Z_{1}^{2} - \alpha )P\left( {1 - P} \right)}}{{i^{2} }} $$

where Z_1_ − $\alpha$ = the normal distribution value = 1.96, P = Relative prevalence of helminths in the region = 15.2%^23^ and $i$ = precision (sampling error) = 0.05

### Data collection

Sociodemographic data was collected using a pretested structured questionnaire from participants and this was used to determine the risk factors. Only participants who could either read or write and understood the questions in the questionnaire were considered for the risk factor. Those who could not read or write but understood the questions were assisted by the researchers to fill out the questionnaire. The questionnaire assessed information on methods of transmission, prevention, and treatments. It also evaluated personal habits with respect to prevention and control methods. Any correct answer was considered as 1 point and any wrong answer was considered as 0. The total score for knowledge and prevention of anaemia was 25 and 10 points respectively. Participants who scored 13 and above for knowledge and 5 and above for anaemia prevention were considered as good knowledge.

Fresh stool samples (minimum being the size of a pigeon egg) were collected from 325 consented participants using clean, dry, wide-mouth sterile specimen bottles with tight covers between 8 am and 2 pm each day. Each stool specimen contained the number, age and, sex of the participant. Stool samples collected were analyzed using the direct smear method at Ebenezer health centre in Melon immediately for the presence of motile larvae before proceeding to the Biological science laboratory of the Faculty of Science, at the University of Bamenda. The parasites were identified using the formol-ether sedimentation technique as described by Christensen et al*.*^24^. Haemoglobin (Hb) concentration was determined using a HemoCue analyser (Hemocue HB 301 analyzer, Ängelholm, Sweden).

The machine was checked daily using the reference microcuvettes as indicated by the manufacturer^25^. Anaemia was classified as severe, moderate, and mild when the Hb < 7 g/dl, 7–9.9 g/dl and 10–10.9 g/dl for the age range of 6–59 months and pregnant women. For the other age groups, it was classified as < 8 g/dl, 8–10.9 g/dl, and 11–11.4 g/dl, for males ≥ 15 years, mild anaemia was classified as 11–11.9 g/dl^6^.

### Data analysis

Data were entered in an Excel spreadsheet and was anonymised. Statistical analysis was done using Statistical Package for Social Sciences (SPSS) version 20 (Chicago, USA). Differences in proportion for age group, sex, occupation, and education were determined using the chi-square test. Mean differences were determined using student t-tests when comparing the means of two groups and ANOVA when comparing the means of more than two groups. While Mann–Whitney U-test was used for geometric mean egg density. Univariate and multivariate analysis was used to explore the relationship between predictors and outcomes of intestinal helminth infections and anaemia. P < 0.05 was considered statistically significant.

## Results

### Characteristics of the study population

A total of 325 participants were recruited for this study with 39.4% (128/325) being males.The mean ± SD age (range) in the study was 31.17 ± 22.9 (0.6–87 years).The 15–30 years age group had the highest number of participants (24.3%, 79/325) while the ≤ 5 years age groups were the least represented (11.1%, 36/325). Equally, the most represented profession was the unemployed 47.1% (153/325). For the level of education the primary level was the most represented group (48.9%, 159/325) while the singles (55.7%, 181/325) were the most represented group as shown in Table 1.

**Table 1:** Socio-demographic characteristics of the study population.

Characteristics	Number of individuals	Frequency (%)
Sex		
Males	128	39.4
Females	197	60.6
Age group(years)		
≤ 5	36	11.1
6–14	73	22.5
15–30	79	24.3
31–45	46	14.2
46–64	51	15.7
≥ 65	40	12.3
Profession		
Civil service	10	3.1
Farming	115	35.4
Trader	47	14.5
Unemployed	153	47.1
Educational level		
Primary	159	48.9
Secondary	99	30.5
University	31	9.5
Informal	36	11.1
Marital status		
Married	107	32.9
Single	181	55.7
Widow(er)	37	11.4

### Prevalence of intestinal helminths in the study population

**Figure 2:**
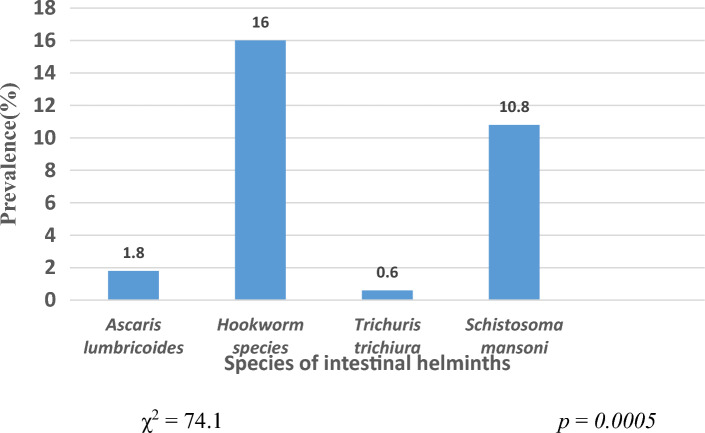
Prevalence of the various species of intestinal helminths in the study area.

Out of the 325 participants, 24.6% (80/325) were infected with one or more parasites. Hookworm species recorded the highest prevalence of 16% (52/325) while *Trichuris trichiura* had the lowest prevalence (0.6%, 2/325). There was a significant difference between the different species of intestinal helminths (P = 0.0005) (Fig. 2). A total of 15 (4.6%) participants had multiple infections: *T. trichiura* and hookworm (2; 0.61%) and hookworm and *S. mansoni* 13/325 (4%).

### Association of age, sex, and occupation on the distribution of intestinal parasitic species

Males were less infected 21.9% (28/128) compared to females 26.4% (52/197). Males equally had a lower prevalence of hookworm 20(15.6%) and *S. mansoni* 11(8.6%) although the differences were not statistically significant (p > 0.05). With regards to age group, participants of the ≤ 5 years age group had the highest prevalence (36.1%, 13/36) while the least was recorded in the 15–30 years age group (13.9%, 11/79). However, following the prevalence of the different parasitic species, 46–64 years had the highest prevalence of *A. lumbricoides* 4(7.8%) and *S. mansoni*, 9 (17.6%), while the ≤ 5 years age group had the highest prevalence of hookworm infection (30.6%, 11/36). The prevalence with respect to age group showed no significant difference (p = 0.055), however, looking at the individual parasites a significantly high prevalence was seen in persons with *A. lumbricoides* infection (p = 0.005) (Table 2).

**Table 2:** Prevalence of intestinal parasitic species based on sex and age and occupation.

Parameters	Number examined	Number infected n (%)	Infection based on parasitic species			
			*A. lumbricoides* n (%)	Hookworms n (%)	*T. trichiura* n (%)	*S. mansoni* n (%)
Sex						
Males	128	28 (21.9)	3 (2.3)	20 (15.6)	2 (0.6)	11 (8.6)
Females	197	52 (26.4)	3 (1.5)	32 (16.2)	0 (0.0)	24 (12.2)
Total	325	80 (24.6)	6 (1.8)	52 (16)	2 (0.6)	35 (10.8)
χ^2^ p-value		0.855 0.355	0.289 0.591	0.022 0.882	3.097 0.078	1.040 0.308
Age group (years)						
≤ 5	36	13 (36.1)	0 (0.0)	11 (30.6)	0 (0.0)	6 (16.7)
6–14	73	14 (19.2)	0 (0.0)	9 (12.3)	0 (0.0)	5 (6.8)
15–30	79	11 (13.9)	0 (0.0)	8 (10.1)	1 (1.3)	5 (6.3)
31–45	46	11 (23.9)	0 (0.0)	7 (15.2)	0 (0.0)	6 (13.0)
46–64	51	17 (33.3)	4 (7.8)	9 (17.6)	0 (0.0)	9 (17.6)
≥ 65	40	14 (35.0)	2 (5.0)	8 (20.0)	1 (2.5)	4 (10.0)
χ^2^ p-value		10.845 0.055	16.719 0.005	6.296 0.279	4.145 0.529	6.874 0.230
Occupation						
Civil servants	10	1 (10.0)	0 (0.0)	1 (10.00)	0 (0.0)	1 (10.0)
Farming	115	29(25.2)	4 (3.5)	14 (12.2)	0 (0.0)	13 (11.3)
Traders	47	15 (31.9)	2 (4.3)	11(23.4)	1 (2.1)	7(14.9)
Unemployed	153	35 (22.9)	0 (0.0)	26 (17.0)	1 (0.7)	14 (9.2)
χ^2^ p-value		2.773 0.428	6.262 0.100	3.550 0.314	2.535 0.469	1.290 0.732

*n* proportion of the population infected.

Occupational-related prevalence showed that the most infected profession was traders (39.1%, 15/47), and the least was seen in civil servants (10.0%, 1/10) as shown in Table 2. Similarly, traders recorded the highest prevalence of all the different parasites identified. These differences were not significant (p > 0.05)..

### Distribution of parasites type and density among the study population

The geometric mean egg density was 77epg and ranged from 20 to 560epg of faeces. Males had an insignificant (p = 0.779) higher intensity (86 epg) of infection than females as shown in Table 3. Age-related intensity of infection showed that the highest load (540 epg) was in the ≤ 5 years age group and the least parasitic load (20epg) was in the 15–30 years age group. Participants who were infected with *S. mansoni* recorded the highest disease burden. However, age and sex-related geometric mean egg density (GMED) showed no significant difference (p > 0.05).

**Table 3:** Distribution of disease burden based on sex and age among the study population.

Parameters	*Ascaris lumbricoides*		Hookworms		*T. trichiura*		*S. mansoni*		Total
	Cases	GMED in epg (Range)	Cases	GMED in epg (Range)	Cases	GMED in epg (Range)	Cases	GMED in epg (Range)	Overall GMED in epg (Range)
Sex									
Males	3	33 (24–240)	20	132 (20–504)	2	24 (24–552)	13	99 (24–420)	86(20–552)
Females	3	17 (24–96)	32	122 (48–480)	0	0	22	92 (24–560)	61(24–560)
Total	6	24 (24–240)	52	126 (20–504)	2	24 (24–52)	35	95 (24–560)	77(20–560)
Statistical analysis		*p* = 1.000		*p* = 0.611		*p* = 1.000		*p* = 0.466	*p* = 0.779
Age group (years)									
≤ 5	0	0	10	248 (96–504)	0	0	6	201 (24–560)	131(24–560)
6–14	2	24 (24–24)	9	100 (20–192)	0	0	5	105 (48–196)	53(20–192)
15–30	0	0	9	123 (48–240)	1	552 (552–552)	7	70 (24–506)	56(24–552)
31–45	0	0	7	82 (48–192)	0	0	5	46 (24–96)	52(24–192)
46–64	4	113 (48–240)	9	118 (72–480)	0	0	8	100 (24–420)	77(24–560)
≥ 65	0	0	8	120 (72–240)	1	24 (24–24)	4	98 (24–144)	75(24–240)
Statistical analysis		*p* = 0.406		*p* = 0.331		*p* = 0.056		*p* = 0.063	*p* = 0.138

*GMED* Geometric mean egg density, *epg* Eggs per gram of stool.

### Variation of mean haemoglobin concentration levels and prevalence of anaemia based on parasitic species

**Figure 3:**
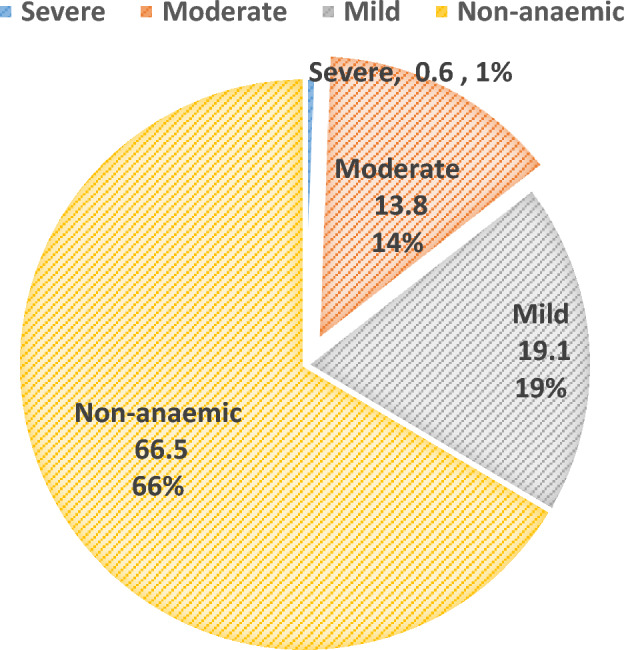
Assessing anaemia prevalence within the study population.

Results from the present study revealed that 33.5% (109/325) of the total participants presented with anaemia. The majority 62(19.1%) of the participants had mild anaemia while only 0.6% (2/325) showed severe anaemia (Fig. 3).

**Figure 4:**
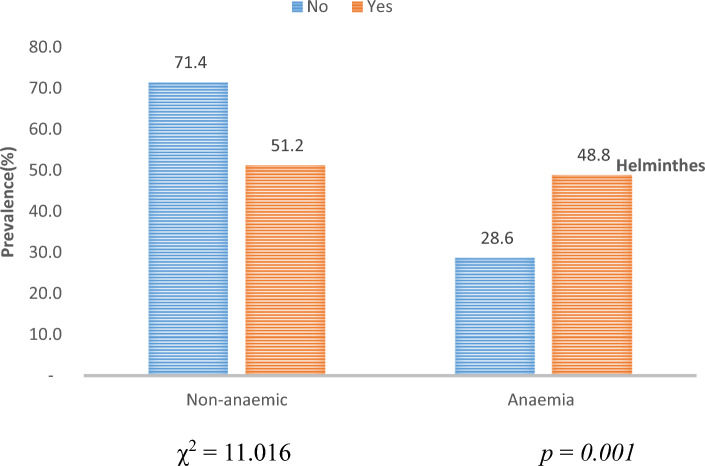
Assessing anaemia prevalence between infected and non-infected participants.

The prevalence of anaemia was significantly higher in those who had one or more parasites (48.8%, 39/80:* p* = 0.001) compared to those that were negative (Fig. 4).

The mean (± SD) of haemoglobin was 12.87(± 1.89) g/dl and ranged from 6 to 14.8 g/dl. The mean ± SD of haemoglobin was significantly lower in participants who were infected (12.10 ± 1.8, p = 0.0001) compared to their negative counterparts (13.63 ± 1.9 g/dl). In Table 4, the mean haemoglobin level was highest among participants infected with *A. lumbricoides* (12.6 g/dl) and least in patients infected with hookworm (11.7 g/dl). However, this difference was not significant (p = 0.614). Out of the 39 infected participants who were anaemic, 23.1% (9/39) of them had multiple infections while 76.9% (30/39) had single infections. Anaemia was significantly higher amongst patients infected with hookworm (53.8%, 28/52: p < 0.05) and lowest in those with *T. trichiura* (0.0% 0/2). All cases of severe anaemia were reported from participants infected with intestinal helminths.

**Table 4:** Variation of mean haemoglobin concentration levels and prevalence of anaemia based on parasitic species.

Intestinal helminths	Number infected	Mean ± SD HB levels (g/dl)	Anaemia prevalence n (%)	Anaemia severity			Statistical analysis	
				Mild n (%)	Moderate n (%)	Severe n (%)	χ^2^	P value
*A. lumbricoides*	6	12.6 ± 1.7	3 (50)	1 (33.33)	2 (66.6)	0 (0.0)	1.982	0.576
Hookworms	52	11.7 ± 1.8	28 (53.84)	12 (42.85)	14(50)	2(7.1)	22.506	0.0001
*T. trichiura*	2	12.1 ± 0.1	0 (00.0)	0 (0.0)	0 (0.0)	0 (0.0)	1.016	0.797
*S. mansoni*	35	11.8 ± 2.1	17 (48.57)	6 (35.29)	9 (52.94)	2 (11.76)	21.972	0.0001
Statistical analysis		F = 14.23	χ^2^ = 11.01					
		P = 0.614	P = 0.001					
Multiple infections								
Hookworms/*T. trichiura*	2	11.0 ± 2.4	0(0.0)	0(0.0)	0(0.0)	0(0.0)		
Hookworms/*S. mansoni*	13	10.8 ± 1.1	9(69.2)	2(22.2)	5(55.6)	2(22.2)		

*HB Haemoglobin.

### Risk factors associated with intestinal helminths

Risk factors were evaluated in 268(82.5%) participants who could either read or write and understood the questions on the questionnaire. In the socio-demographic bivariate analysis, our data revealed that the prevalence of intestinal helminths was significantly highest in older subjects ≥ 65 years (35.0%, 14/40: p = 0.045) and insignificantly higher in females (25.7%, 43/167: p > 0.05), those who did not go to school 6(40.0%; 6/15), traders (31.9%; 15/47) and those who were married 33(31.43%; 33/105). Assessing knowledge of participants and their influence on living habits showed that those who had poor knowledge of the parasites had a higher prevalence of 27.8% (55/198), those who did not usually deworm after 3 months 29.9% (60/201), geophagia 25(34.7%), participants who used toilet made of sticks 64.0% (16/25), and participants who eat roadside food 45(25.4%) all had significantly higher prevalence (p > 0.05). On the other hand, participants who sometimes washed their fruits or vegetable had a prevalence of 37.2% (15/40), those who do not wash their hands after the toilet 6(35.3%), those that walk barefoot at times 8(26.7%) recorded the highest prevalence though it was insignificant (p > 0.05). Considering some common clinical symptoms within the past 3 months, parasitic infections were significantly (p = 0.022) higher (55; 28.1%) in patients without stomach or abdominal pains and significantly (p = 0.003) higher (27, 36.0%) in patients with anaemia.

All factors that were significant in the univariate analysis were used in the multivariable analysis. After adjusting all the confounders in the multivariate regression model, it was found that the age group 31–45 years [AOR = 3.42; 95% CI; 1.05–11.21, P = 0.042] and > 65 years [AOR = 6.21; 95% CI; 1.75–12.47, P = 0.005), poor knowledge [AOR = 0.41; 95% CI; 0.67–6.07, P = 0.043], not deworming regularly [AOR = 0.70, 95% CI 0.19–0.29, P = 0.0001], use of mud floor toilet [AOR = 6.18; 95% CI; 1.61–23.79, P = 0.008], toilets made of sticks [AOR 16.5, 95% CI 4.24–64.31, P = 0.0001], those without stomach or abdominal pains (AOR = 0.22; 95% CI; 0.07–0.67, P = 0.04) and anaemia [AOR = 3.41; 95% CI; 1.33–817 P = 0.011] were identified as risk factors for intestinal parasitic infections as shown in Table 5.

**Table 5:** Assessment of risk factors associated with intestinal helminths.

Predictors	Number examined (%)	Number infected (%)	COR(95%CI)	p-value	AOR(95%CI)	p-value
Age in years				0.045		
< 15	52(19.4)	9(17.3)	R			
15–30	79(29.5)	12(15.2)	1.75(0.70–4.4)		3.40(0.93–12.47)	0.065
31–45	46(17.2)	11(23.9)	2.79(1.20–6.51)		3.42(1.05–11.21)	0.042
46–65	51(19.0)	17(33.3)	1.17(0.45–3.01)		0.90(0.26–3.11)	0.87
≥ 65	40(14.9)	14(35.0)	3.01(1.23–7.35)		6.21(1.75–12.47)	0.005
Gender				0.26		
Female	167(62.3)	43(25.7)	1.34(0.39–1.29)			
Male	101(37.7)	20(19.8)	R	–		
Level of education				0.23		
Primary	124(46.30)	29(23.4)	0.45 (0.15–1.39)			
Secondary	98(36.60)	24(24.5)	0. 49(0.15–1.56)			
University	31(11.6	4(12.9)	0.22 (0.05–96)			
None	15(5.60)	6(40.0)	R			
Occupation				0.22		
Civil servant	10(3.70)	1(10)	4.21 (0.48–36.40)			
Farmers	115(42.90)	29(25.2)	2.07 (0.24–17.45)			
Traders	47(17.50)	15(31.9)	3.03 (0.36–24.99)			
Unemployed	96(35.80)	18(18.8)	R	–		
Marital status				0.29		
Married	105(39.20)	33(31.4)	2.44(0.78–16.52)			
Single	127(47.40)	21(16.5)	3.76(0.48–18.47)			
Widow(er)	36(13.40)	9(25.0)	R			
Knowledge on intestinal helminths				0.007		
Poor	198(73.9)	55(27.8)	0.33(0.15–0.80)		0.41(0.67–6.07)	0.043
Good	70(53.0)	8(11.4)	R		R	
Wash fruits and vegetables before eating				0.33		
No	7(2.6)	2(28.5)	2.88(0.31–26.5)			
Sometimes	40(14.9)	15(37.2)	1.71(0.20–14.5)			
Yes	221(8.25)	46(20.8)	R			
Wash your hands after the toilet				0.07		
No	17(6.3)	6(35.3)	0.40(0.14–1.20)			
Sometimes	108(40.3)	31(28.7)	0.74(0.25–2.17)			
Yes	143(53.4)	26(18.2)	R			
Deworm after three months				0.0001		
No	201(75.0)	60(29.9)	0.16(0.03–0.36)		0.07(0.19–0.29)	0.0001
Yes	67(25.0)	3(4.5)	R		R	
Geophagia				0.010		
No	196(73.2)	38(19.4)	2.21(1.21–4.03)		2.19(0.93–5.19)	0.074
Yes	72(26.8)	25(34.7)	R			
Type of toilet				0.000		
Mud	17(6.3)	10(58.8)	8.33(3.44–20.37)		6.18(1.61–23.79)	0.008
Sticks	25(9.3)	16(64.0)	0.38(0.84–1.65)		16.5(4.24–64.31)	0.000
Cement	199(74.3)	35(17.6)	6.69(2.29–18.79)		0.31(0.49–1.93)	0.209
Water system	27(10.1)	2(7.4)	R		R	
Water source				0.02		
Spring	27(10.1)	11(40.7)	0.82(0.29–2.29)		0.97(0.24–4.02)	0.976
Stream	36(13.4)	13(36.1)	0.33(0.14–0.77)		0.36(0.12–1.14)	0.083
Tap	189(70.5	35(18.5)	0.48(0.12–1.93)		0.31(0.04–2.33)	0.260
Well	16(6.0)	4(25.0)	R		R	
Walk barefooted				0.55		
No	96(35.8)	19(19.8)	1.47(0.57–3.81)			
At times	30(11.2)	8(26.7)	1.37(0.73–2.58)			
Yes	142(53.0)	36(25.4)	R		R	
Eat roadside food				0.011		
No	83(31.0)	13(15.7)	8.97(1.90–42.23)		8.45(0.95–75.14)	0.056
Sometimes	8(3.0)	5(6.25)	1.83(0.092–3.62)		2.28(0.86–6.08)	0.098
Yes	177(66.0)	45(25.4)	R			
Fever				0.806		
No	214(79.9)	51(23.8)	0.91(0.45–1.86)			
Yes	54(20.1)	12(22.2)				
Stomach or abdominal pain				0.022		
No	196(73.1)	55(28.1)	0.32(0.14–0.72)		0.22(0.07–0.67)	0.04
Yes	72(26)	8(11.1)				
Headache				0.34		
No	195(72.8)	51(26.2)	2.52 (1.35–4.44)			
Yes	73(27.2)	12(16.4)				
Anemia				0.003		
No	193(72.0)	36(18.7)	5.08(1.72–15.02)		3. 40(1.33–8.71)	0.011
Yes	75(28.0)	27(36.0)				

*R Reference; *COR* crude odds ratio; *AOR* adjusted odds ratio.

### Assessing risk factors associated with anaemia

Anaemia prevalence was highest in the age group of < 15 years (53.8%; 28/52) and least in those aged above 64 years (15.0%; 6/40). Similarly, the prevalence of anaemia was higher in females (31.7%; 52/167), illiterates (40.0%; 6/15), farmers (33.9%, 39/115), widow(er)s (44.4%; 16/36), geophagia participants (45.8%; 33/72), those infected with any of the parasites [*A. lumbricoides:* (33.3%, 2/6), hookworm (46.2%, 18/39), *T. trichiura* (50.0%, 1/2), *S. mansoni* (44.4%, 12/27], those with multiple parasitic infections (45.5% 5/11). A significant difference was observed among the age group, occupation, marital status, geophagia participants, those infected with hookworm and* S. mansoni,* and those with multiple infections (P < 0.05). Only variables that were significantly associated with anaemia in the binary logistic regression were considered for the multivariate analysis. After adjusting for confounders, the risk of having anaemia was observed more in the age group < 15 years [AOR, 95% CI 2.58(1.09–6.11), P = 0.03] with an increased risk of 5.8%. The risk was also 6.9 times higher among geophagia participants. Anaemia was more prevalent among participants infected with hookworm with a 2.6% increased risk [AOR, 95% CI 3.59(1.65–7.87), P = 0.001], and in *S. mansoni* the risk was increased by 7.2% [AOR, 95% CI 1.72 (1.16–3.41), P = 0.021]. Moreover, the risk increased 7.6 times in participants with multiple infections [AOR, 95% CI 1.76(1.04–2.88), P = 0.03] (Table 6).

**Table 6:** Assessment of risk factors associated with anaemia.

Predictors	Number examined (%)	Presence of anaemia (%)	COR (95%CI)	p-value	AOR (95%CI)	p-value
Age in years				0.0001		
< 15	52(19.4)	28(53.8)	0.46(0.18–1.19)		2.58(1.09–6.11)	0.03
15–30	79(29.5)	22(27.8)	0.80(0.35–1.79)		1.40(0.44–4. 50)	0.57
31–45	46(17.2)	7(15.2)	1.02(1.45–6.29)		0.69(0.21–2.38)	0.56
46–64	51(19.0)	12(23.5)	0.17(0.16–1.23)		0.78(0.21–3.00)	0.72
65 +	40(14.9)	6(15.0)				
Gender				0.296		
Females	167(62.3)	52(31.7)	1.33 (0.77–2.30)			
Males	101(37.7)	23(22.8)	**R**			
Education				0.42		
Primary	124(46.30)	37(29.8)	0.81(0.26–2.56)			
Secondary	98(36.60)	25(25.5)	0.84(0.26–2.67)			
University	31(11.60)	7(22.5)	0.38(0.91–1.62)			
None	15(5.60)	6(40.0)	**R**			
Occupation				**0.0002**		
Civil servant	10(3.70)	1(10)	0.79 (0.16–4.02)		0.75(0.14–4. 19)	0. 75
Farmers	115(42.90)	39(33.9)	1.37(0.25–7.37)		0.99(0.17–5.89)	0.99
Traders	47(17.50)	14(29.8)	3.11(0.63–15.42)		1.06(0.16–6.86)	0.95
Unemployed	96(35.80)	21(21.9)				
Marital status				**0.0003**		
Married	105(39.20)	31(29.5)	2.76(1.50–5.04)		1.78(0.70–4.50)	0.70
Single	127(47.40)	28(22.0)	0.68(0.23–1.98)		0.73(0.22–2.36)	0.23
Widow	36(13.40)	16(44.4)	R		**R**	
Geophagia				0.0001		
No	196(73.2)	42(21.4)	2.10(0.75–4.72)		3.69(1.91–9.33)	0.004
Yes	72(26.8)	33(45.8)	R			
*Ascaris lumbricoides*						
No	262(97.8)	73(27.9)	2.63(0.52–13.37)	0.25		
Yes	6(2.2)	2(33.3)	**R**			
Hookworm						
No	229(85.4)	57(24.9)	2.58(1.28–5.19)	0.008	3.26(1.49–7.11)	**0.003**
Yes	39(14.6)	18(46.2)	**R**			
*Trichuris trichiura*						
No	266(99.3)	74(27.8)	*			
Yes	2(0.7)	1(50.0)	**R**			
*Schistosoma mansoni*						
No	241(89.9)	63(26.1)	1.96 (1.57–3.14)	0.001	1.72 (1.16–3.41)	0.021
Yes	27(10.1)	12(44.4)	**R**			
Multiple infections						
No	257(95.9)	71(27.6)	1.57 (1.01–3.28)	**0.044**	1.76(1.04–2.88)	0.030
Yes	11(4.1)	5(45.5)				
Knowledge of intestinal helminths						
Poor	198 (73.9)	57(28.8)	1.04 (0.57–1.90)	0.88		
Good	70 (53.0)	18(25.7)	**R**			
Prevention of anaemia						
No	12(4.5)	3(25.0)	0.85 (0.22–3.23)	**0.81**		
Yes	256(95.5)	72(28.1)				

Significant values are in [bold].

*Positive entry < 5 *R Reference.

## Discussion

Studying infection prevalence and morbidity of intestinal helminths and associated predictors of risk factors is a primary objective to identify high-risk communities and select appropriate intervention mechanisms. This study aimed to determine the prevalence, anaemia status, and risk factors due to intestinal helminths in a cross-section of the population of Melong which is a rural area, a place where travellers stop to buy food and fruits and also ease themselves before they continue their journey.

The results of this study showed that the overall prevalence of intestinal helminths was 24.6%. In our opinion, this prevalence is on the high side. This might probably be because Melong is a crossroad where passengers from the Northwest, Southwest, Littoral, and Western regions always stop to rest, eat and buy items thereby urinating and defecating around the environment which is a common practice especially at night. Equally most inhabitants of Melong being a rural setting depend on streams, springs, and wells for their daily domestic water supply, thus increasing their chances of getting infected. Moreover, the hot and humid climate of Melong accompanied by poor sanitation might also be associated with the infection rate. This Climate favours the snail vectors reproduction and the hatching of hookworm and *Trichuris* eggs.

Similar findings were observed in Buea by Mbuh et al*.*^11^, who had a prevalence of 28.1%, and in Ethiopia with a prevalence of 24.7%^19^. However, the prevalence was higher when compared to that of Kuete et al*.*^23^ who had a prevalence of 15.2% and of a study carried out in Kenya^26^ which observed a prevalence of 13.8%. A higher prevalence of 43.6–44.34% has been reported in other studies^8^. These differences might also be due to differences in socio-economic conditions of the study population and individual behavioural habits, sanitation, presence of water, and environmental conditions such as humidity, temperature, and lack of sensitivity to diagnostic methods of the different intestinal helminths.

The most prevalent intestinal helminth was hookworm with a 16.0% prevalence, followed by *S. mansoni* with 10.8%. The high prevalence of hookworm in the present study is similar to other studies conducted in Cameroon^8^ and Ethiopia^19,21^. Melong being a stopover for people to eat when travelling leads to open defecation, thus contaminating the environment. Furthermore, the majority of the participants (53%) had the habit of walking barefooted and were farmers (42.9%). These two factors predispose individuals to hookworm infection. A high prevalence of *S. mansoni* seen in this study is expected since the climate of Melong favours the development of snail intermediate host species coupled with the fact that Melong is a rural setting with minimal hygienic conditions and most of the inhabitants are farmers and traders that depend on streams and springs for their daily activities thereby resulting in them coming in contact with soil-transmitted helminths and *Schistosoma*. Higher records of *Schistosoma* have equally been observed in a study carried out by Thiong’o et al*.*^27^ and Pone et al*.*^28^ in Kenya. These findings might indicate a need for epidemiological updates on *S. mansoni* in the Littoral region of Cameroon.

An overall co-infection prevalence of 4.6% was recorded in this study, with hookworm**/***S. mansoni* co-infection dominating. Higher co-infection rates have been reported with *Ascaris lumbricoide*s and hookworm co-infection being the most common. Multi-parasitic infections are usually common because most parasites share similar transmission routes which are also associated with the level of environmental contamination, lack of knowledge of the parasitic infections, and socioeconomic factors. As shown in this study, the lack of knowledge about the disease was also a predictor of infection.

Sex-related prevalence showed that females were more infected (26.4%) than males (21.9%). Also, intensity of infection was higher in females than males. This might be due to their daily activities in Melong. Women are more exposed to farming activities and usually tend to neglect washing their hands before eating. Secondly, in the univariate analysis, geophagia was seen as a predictor although it shows a trend in the multivariate analysis. Previous studies have shown that geophagia is a common practice among females and has been associated with high rates of helminth infections^29,30^. On the contrary, Payne et al*.*^31^ and Tchinde et al*.*^4^ reported a higher prevalence in males in the western region of Cameroon. This could be a result of cultural differences whereby in the western region, farming activities are being carried out mostly by males.

Participants of the ≤ 5 years age group were the most prevalent (36.1%), with hookworm taking the lead, similar to the findings of Payne et al*.*^31^ from Cameroon and Nmor et al*.*^32^ from Nigeria. This might be because young children play a lot with the soil and have the habit of putting their hands into their month without washing them. Thus exposing them to infection. In addition, it has been reported that immunological responses vary with age^32^ thus the immune system in younger children has not yet reached maturity, resulting in their high vulnerability to infections. This also accounts for the high anaemia prevalence among the younger age group seen in this study.

Occupational-related prevalence showed that traders were the most infected group (31.9%) which could be attributed to daily social activities that expose them to infections. Educational prevalence showed that those with non-formal education were the most infected. Health education is one of the most powerful tools in controlling helminth infections thus ignorance of the infection routes of these diseases due to non-formal education can be very instrumental in the transmission of helminth infections. This further goes a long way to indicate that the adult community members of Melong may be a source of continued infection in school-age children since the deworming program targets only school-age children, hence hindering the control programmes. Similar findings were observed in the coastal region of Kenya^33^, which showed that most control programmes for schistosomiasis and STH infections often target school-age children only, leaving out the rest of the community members who are equally infected and may act as reservoirs for transmission and a source of re-infection to the school-age children.

In this study, we found out that the use of mud floors and toilets made of sticks, not deworming regularly, poor knowledge of intestinal parasites, and those who presented with stomach or abdominal pains increased the risk of infection with intestinal parasitic infection. All these factors are very crucial contributing factors to the transmission of helminths in any given location. Stomach or abdominal pains is a common symptom associated with intestinal helminths.

The use of mud floors and toilets made of sticks as risk factors is similar to records in other towns in the Littoral region of Cameroon^8,23^. There is a high chance that such toilets cannot be properly cleaned and so serve as a route of disease transmission. However, a study from the Northwest Region of Cameroon by Ntonifor et al.^12^ reported the use of water system toilets as a risk factor due to the scarcity of water in that study area which resulted in most of the inhabitants practicing promiscuous defecation. A high prevalence seen in those who presented with stomach or abdominal discomfort accentuates the fact that intestinal helminths are known to cause abdominal and stomach pains.

The prevalence of anaemia was 33.5% while anaemia associated with intestinal helminth infections was 9.2%. A higher prevalence of 17.5% has been recorded in another study by Ntonifor et al.^12^. The difference can be attributed to the different study populations as well as the sensitivity of the techniques used in the determination of haemoglobin levels. The high prevalence of anaemia in parasitic infections is associated with blood loss leading to iron deficiency anaemia, loss of appetite, competition for micronutrients, as well as malabsorption of nutrients. metabolism. Hookworms reside in the small intestines of infected individuals where they attach themselves to the villi and feed on host blood. On the other hand, the lateral spines in the eggs of schistosomes are responsible for piecing the intestinal walls this causing blood loss in infected individuals.

Moreover, the high prevalence (28.6%) of anaemia seen among those who were not infected indicates that the cause of anaemia is multi-factorial and might be due to bacteria (tuberculosis), viruses (human immunodeficiency virus), protozoa (malaria, leishmaniasis) or iron deficiencies^17,19,20^.

In this study geophagia participants were at risk of anaemia. Similarly, the prevalence of intestinal helminths were higher in geophagia participants compared to non-geophagia participants. This is in line with studies carried out elsewhere^29,30^. Eating soil has been associated with decreased absorption of nutrients due to the physical damage of the intestinal mucosa. We equally observed that participants infected with hookworm, *Schistosoma,* and multiple infections were more likely to develop anaemia contrary to *Ascaris lumbricoides* as reported by Mengist et al.^19^. Hookworms ingest blood and damage the intestinal wall causing bleeding thus contributing to anaemia by causing blood loss directly through ingestion and mechanical damage of the mucosa, and indirectly, by affecting the supply of nutrients necessary for erythropoiesis thus accounting for the highest prevalence in hookworm infested participants. Anaemia in patients harbouring *S. mansoni* is due to the spines of schistosomes that pierce and wound infected individuals thereby causing blood to flow out through the wounds resulting in anaemia. Lack of Knowledge though not identified as a risk factor is a call for concern as it is a major reason why people don’t use complementary health approaches observed in the study, since illiterates recorded the highest prevalence of helminths and anaemia, suggesting that community education needs to be intensified in the study area.

## Conclusions

This study revealed a significant burden of gastrointestinal parasites and anaemia in a rural community of Melong-Cameroon. Hookworm and *S. mansoni* infections were shown to be significantly associated with anaemia. Therefore, detection of helminth infections and haemoglobin levels should be done simultaneously to minimize the risk of anaemia in infected participants. We recommend improved health education and awareness relating to hygienic practices, better sanitation, and a supply of pipe-borne water to help decrease these parasitic infections in endemic communities. Regular deworming should be encouraged by the government for both adults and children.

### Strength

The study identified for the first time risk factors associated with helminthiasis and anemia in Melong Littoral Region of Cameroon. These factors will be used by policymakers to implement preventive methods that will be beneficial to the inhabitants of Melong.

### Limitations

The relative abundance and transmission dynamic on snails was not done to determine if the vectors are available in the study area. Secondly, we could not assess other factors associated with anemia.

### Research perspective

Identification of different vectors of schistosomiasis should be carried out in the study area. Secondly other factors such as nutritional status, and diagnosis of other pathogens (bacteria, malaria, etc.) associated with anaemia should be carried out in this area.

## Abbreviations

*A.*: *Ascaris*
epg: Eggs per gram of stool
*S.*: *Schistosoma*
STHs: Soil-transmitted helminths
*T.*: *Trichiura*
